# Purification and partial characterization of LdtP, a cell envelope modifying enzyme in *Liberibacter asiaticus*

**DOI:** 10.1186/s12866-018-1348-8

**Published:** 2018-11-29

**Authors:** Janelle F. Coyle, Fernando A. Pagliai, Dan Zhang, Graciela L. Lorca, Claudio F. Gonzalez

**Affiliations:** Department of Microbiology and Cell Science, Genetics Institute and Institute of Food and Agricultural Sciences, University of Florida, Gainesville, FL USA

**Keywords:** Citrus greening, LdtP, *Liberibacter asiaticus*, L,D-transpeptidase, Esterase, Lipopolysaccharide, Peptidoglycan, Cell envelope

## Abstract

**Background:**

The aggressive spread of *Liberibacter asiaticus*, a bacterium closely associated with citrus greening, has given rise to an acute crisis in the citrus industry, making it imperative to expand the scientific knowledge base regarding *L. asiaticus*. Despite several endeavors to culture *L. asiaticus*, this bacterium has yet to be maintained in axenic culture, rendering identification and analysis of potential treatment targets challenging. Accordingly, a thorough understanding of biological mechanisms involved in the citrus host-microbe relationship is critical as a means of directing the search for future treatment targets. In this study, we evaluate the biochemical characteristics of CLIBASIA_01175, renamed LdtP (L,D-transpeptidase). Surrogate strains were used to evaluate its potential biological significance in gram-negative bacteria. A strain of *E. coli* carrying quintuple knock-outs of all genes encoding L,D-transpeptidases was utilized to demonstrate the activity of *L. asiaticus* LdtP.

**Results:**

This complementation study demonstrated the periplasmic localization of mature LdtP and provided evidence for the biological role of LdtP in peptidoglycan modification*.* Further investigation highlighted the role of LdtP as a periplasmic esterase involved in modification of the lipid A moiety of the lipopolysaccharide. This work described, for the first time, an enzyme of the L,D-transpeptidase family with moonlighting enzyme activity directed to the modification of the bacterial cell wall and LPS.

**Conclusions:**

Taken together, the data indicates that LdtP is a novel protein involved in an alternative pathway for modification of the bacterial cell, potentially affording *L. asiaticus* a means to survive within the host.

**Electronic supplementary material:**

The online version of this article (10.1186/s12866-018-1348-8) contains supplementary material, which is available to authorized users.

## Background

Citrus greening, or huanglongbing (HLB), is the most formidable and devastating disease to ever affect the citrus industry. Citrus is an essential crop grown in areas ±40° latitude of the equator, with Brazil, China, and the United States as the top citrus producers worldwide [1, 2]. The aggressive spread of HLB has given rise to an acute crisis for citrus growers with no proximately discernible curative control method.

HLB is associated with three species of fastidious, phloem-limited α-proteobacteria: *Liberibacter asiaticus* in Asia and North America, *Liberibacter africanus* in Africa, and *Liberibacter americanus* in Brazil [1]. *L. asiaticus* is the most pathogenic of these species and is closely associated with HLB in the United States [1, 2]. It is transmitted among citrus plants via the feeding activities of the psyllid vector, *Diaphorina citri* [1, 2]. Following infiltration and colonization of the citrus host, the HLB pathogen spreads rapidly to all plant tissues causing yellow shoots, non-symmetrical blotchy mottled leaves, and small, deformed, abnormally colored fruit [2]. Although several treatment methods have been investigated, current HLB management is limited to the control of the psyllid vector and the removal of infected trees [1, 2]. It has been estimated that without intervention, the citrus industry will diminish substantially within 2–10 years in affected regions. As such, it is imperative to develop new antimicrobial therapies against HLB for the preservation of this industry.

Despite several endeavors to culture *L. asiaticus*, this bacterium has yet to be maintained in axenic culture, rendering identification and analysis of potential treatment targets challenging. Additionally, genes encoding toxins or other pathogenicity genes were not identified by an analysis of the *L. asiaticus* genome [3]. However, a genome analysis was successful in detecting only 10 transcription factors in the genome, suggesting that targeted chemical treatment directed at one individual transcription factor may result in pleiotropic effects, thereby reducing viability of the pathogen [4].

LdtR, a member of the multidrug resistance regulator (MarR) family of transcriptional regulators, was functionally characterized as a target for the development of novel *L. asiaticus* treatments [5]. In the *L. asiaticus* genome, the *ldtR* gene is encoded immediately upstream from the *ldtP* gene, which encodes a putative L,D-transpeptidase (LD-TPase). This gene arrangement is highly conserved among all members of the *Rhizobiaceae* family. LdtR acts as a transcriptional activator of both *ldtR* and *ldtP* by binding to the promoter regions of these genes [5]. In a closely-related species, *Sinorhizobium meliloti*, bacteria harboring a disruption in *ldtR* or *ldtP* exhibited reduced tolerance to osmotic stress, as well as a short-cell phenotype attributed to alterations in cell wall architecture. Further, it was established that the activation of transcription of *ldtP* by LdtR is essential for the osmotic stress response, and, specifically, it is predicted that LdtP-mediated remodeling of the bacterial cell wall is vital for adaptation and resistance to osmotic stress. Since the L,D-transpeptidase enzymes are involved in bacterial cell wall crosslinking, allowing an increased resistance to osmotic stress, we decided to analyze the characteristics of the encoded protein.

**Fig. 1:**
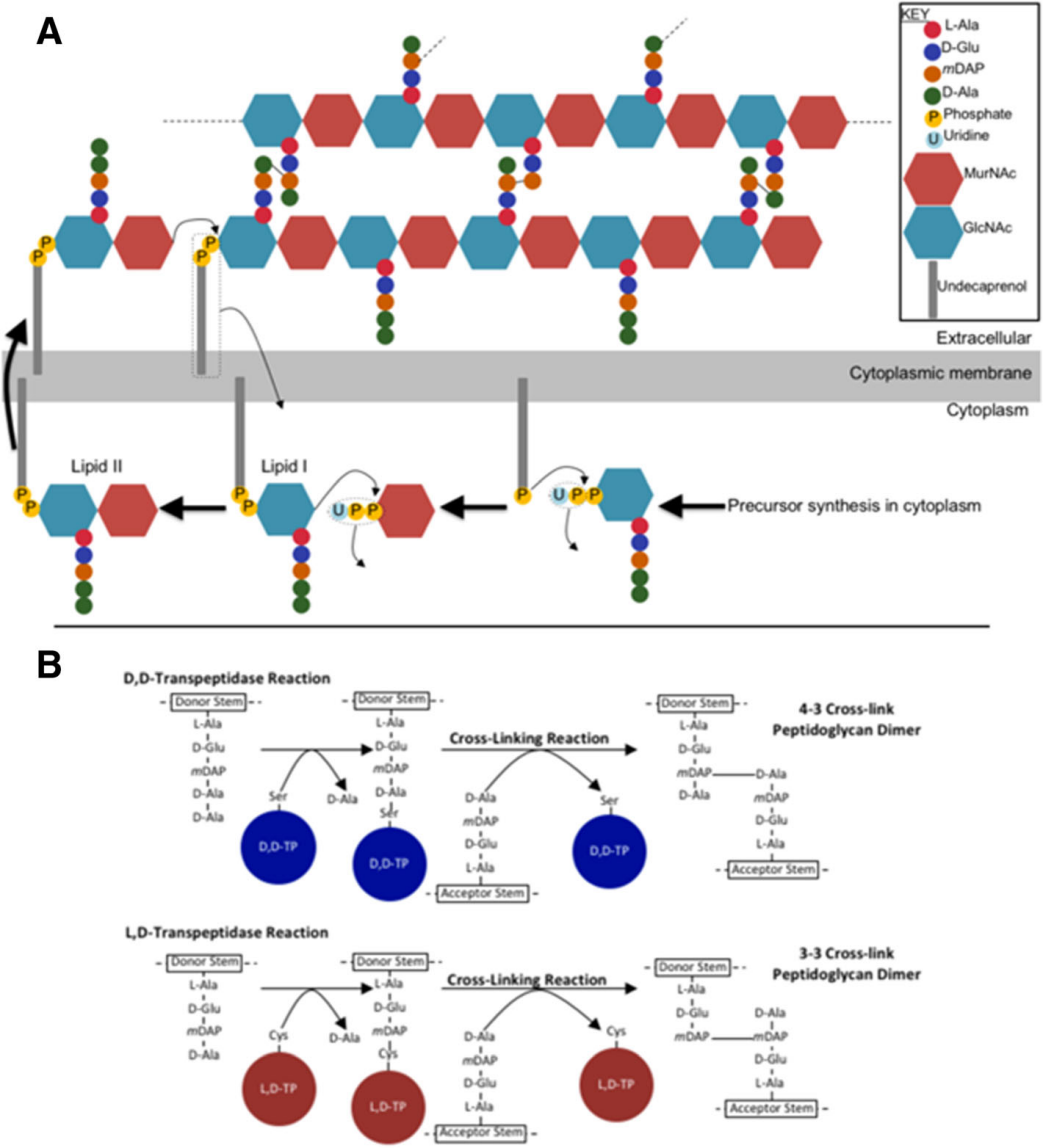
Biosynthesis of the bacterial cell wall. **a** Diagram of the synthesis of the cell wall in a gram-negative bacterium. **b** Mechanism of the cross-linking reaction catalyzed by D,D-TPases and L,D-TPases

LD-TPases are enzymes involved in the biosynthesis and rearrangement of the bacterial cell wall, a durable and flexible peptidoglycan polymer responsible for maintaining cell shape and protecting the cell from environmental stressors [6]. Peptidoglycan is a versatile structure composed of linear glycan chains linked together by peptide cross-bridges, creating a lattice-like mesh [7]. The subunit of peptidoglycan, synthesized in the cytoplasm, is a disaccharide-peptide composed of an N-acetylmuramic acid-N-acetylglucosamine (MurNAc-GlcNAc) dimer linked via a β-1,4-glycosidic bond with a linear pentapeptide stem. This stem consists of L-Ala^1^-D-Glu^2^-*meso*DAP^3^-D-Ala^4^-D-Ala^5^ linked to the MurNAc moiety in most gram-negative bacteria (Fig. 1a). This precursor is flipped across the inner membrane into the periplasm, where glycosyltransferases catalyze bond formation between GlcNAc of the precursor subunit and MurNAc of the growing glycan chain. LD-TPases and D,D-transpeptidases (DD-TPases) are responsible for cross-link formation between peptide stems on adjacent glycan chains, producing a rigid, three-dimensional peptidoglycan sacculus.

The degree and type of peptidoglycan cross-linking varies among bacterial species and even within a species, depending on the growth phase and environmental conditions. The main type of linkage is the 4–3 type generated by DD-TPases, whereas LD-TPases catalyze the formation of the less abundant 3–3 linkages (Fig. 1b). DD-TPases, also known as penicillin-binding proteins, are sensitive to all classes of β-lactam antibiotics and contain an active-site serine residue that facilitates the formation of 4–3 cross-links. These enzymes cleave the terminal D-Ala^5^ from a pentapeptide donor stem adjoining the carbonyl of the resulting D-Ala^4^ to the amine group of *meso*DAP^3^ on an acceptor stem. Alternatively, LD-TPases are insensitive to all classes of β-lactam antibiotics except the carbapenems, contain an active-site cysteine residue, and require the activity of D,D-carboxypeptidases to generate a tetrapeptide donor stem. LD-TPases generate 3–3 cross-links by cleaving the D-Ala^4^ of a tetrapeptide donor stem and linking *meso*DAP^3^ on the donor stem to the *meso*DAP^3^ of the acceptor stem. An increase in the cross-linking activity of LD-TPases induces increased β-lactam resistance, confers protection during the stationary phase of growth, and fortifies the cell wall upon exposure to stressors such as temperature fluctuation, osmotic stress, and nutrient limitation.

The goal of this study was to thoroughly characterize LdtP with bioinformatic techniques and to utilize a genetic approach to observe the phenotypic influence of LdtP overexpression in a strain of *Escherichia coli* lacking all five of the encoded LD-TPases [7]. The *E. coli* quintuple mutant (Δldt5) and *Liberibacter crescens* were used as models in this study because *L. asiaticus* cannot be cultured in the laboratory.

## Results

### Characterization of LdtP

**Fig. 2:**
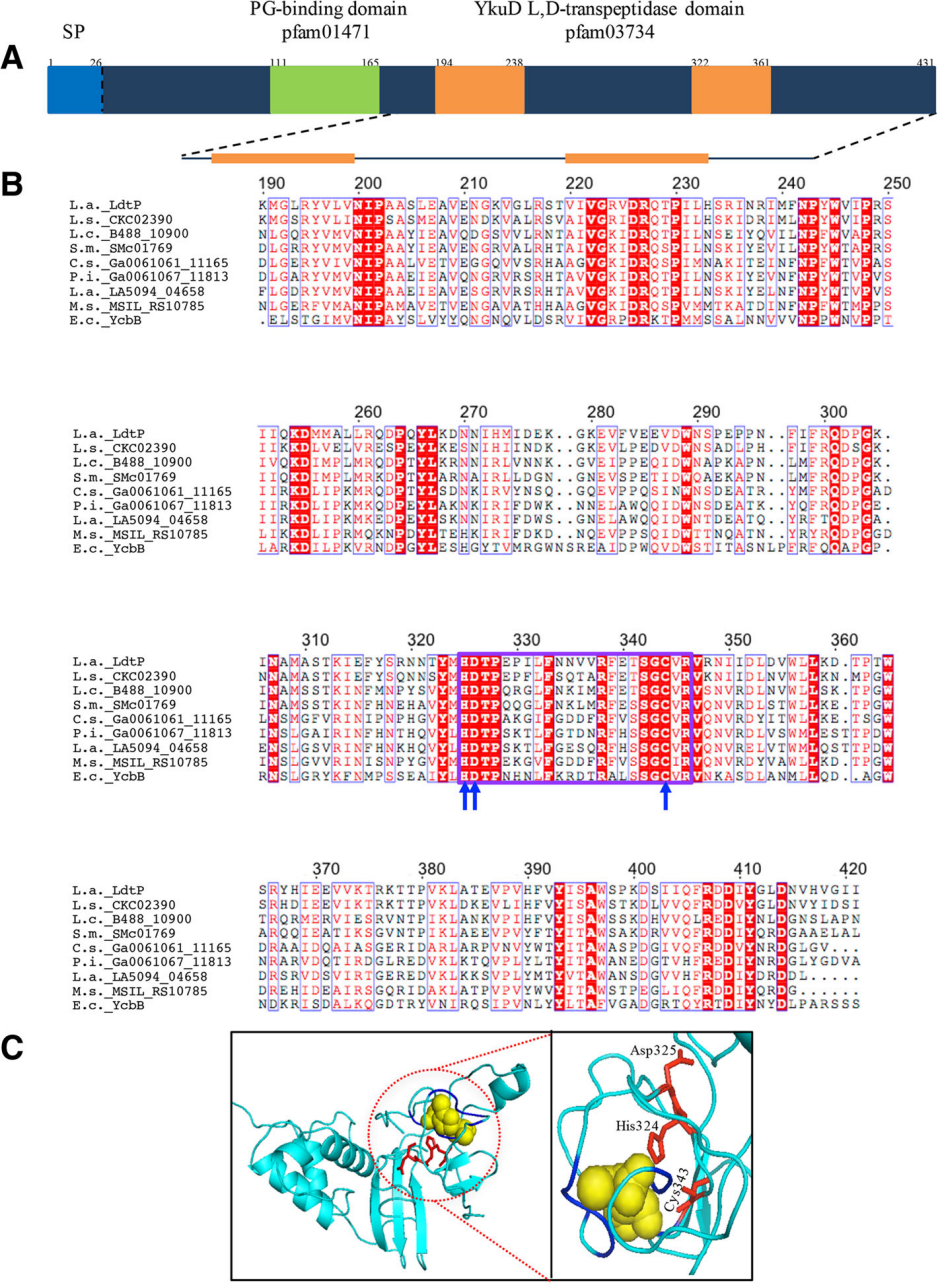
Conserved domains, sequence alignment, and structure of LdtP. **a** The domain arrangement of LdtP according to the NCBI Conserved Domain Database. **b** A partial sequence alignment covering the L,D-transpeptidase catalytic domain (pfam03734) of LdtP and homologous L,D-transpeptidase proteins identified by PSI-Blast. Identical residues are colored white with a red background; columns with higher than 70% equivalent residues considering physico-chemical properties are boxed and colored red with a white background; catalytic residues are denoted by blue arrows; the conserved motif, HXX_14–17_[S/T]HGChN, is boxed in purple. Abbreviations (accession numbers): L.a._LdtP, *Liberibacter asiaticus*, (ACT56823); L.s._CKC_02390, *Liberibacter solancearum* (ADR52227); L.c._B488_10900, *Liberibacter crescens* (AGA65082); S.m._SMc01769, *Sinorhizobium meliloti* (NP_385313); C.s._Ga0061061_11165, *Chelatococcus sambhunathii* (CUA90119); P.i._Ga0061067_11813, *Pannonibacter indicus* (CUB00332); L.a._LA5094_04658, *Labrenzia alba* (CTQ61876); M.s._MSIL_RS10785, *Methylocella silvestris* (ACK51051); E.c._YcbB, *Escherichia coli* (NP_415445). The figure was made using the web server ESPript [47]. **c** A three-dimensional model of LdtP (residues 98–356) predicted by PHYRE2 based on Ldt_Mt1_ from *Mycobacterium tuberculosis* (PDB:4JMX) as the template. Yellow, imipenem; blue, binding pocket; red, catalytic residues (labeled)

Analysis of the *L. asiaticus* str. Psy62 genome revealed a single putative LD-TPase, encoded by *CLIBASIA_01175*, renamed *ldtP.* This gene encodes a hypothetical protein (Accession: ACT56823) composed of 431 amino acids assigned to COG2989 (murein L,D-transpeptidase YcbB/YkuD) with two identified conserved domains: (1) a peptidoglycan-binding domain (pfam01471); and (2) two YkuD L,D-transpeptidase catalytic domains (pfam03734) (Fig. 2a) [8]. Members of the YkuD superfamily are typified by a completely conserved active-site cysteine and histidine residue, as well as a third residue that accepts a hydrogen bond from histidine. According to a PSI-Blast multiple sequence alignment (Fig. 2b), this catalytic triad is composed of Cys343, His324, and Asp325.

The *ldtP* sequence was predicted by the Phobius server to encode an N-terminal signal peptide that directs LdtP to the cytoplasmic membrane for export into the periplasm. There, the mature protein has access to the cell wall and can participate in biosynthesis and rearrangement activities. The signal peptide is cleaved between Ser25 and Lys26 as LdtP is transported into the periplasm [9]. Following cleavage of the signal peptide, LdtP was calculated to have a theoretical molecular mass of 46.5 kDa and a pI of 5.82 [10]. This molecular mass was confirmed following purification of recombinant His-tagged LdtP through SDS-PAGE (Additional file 1: Figure S1).

The three-dimensional structure of LdtP was modeled by the Phyre2 server (Fig. 2c). The template for the construction of this model was an LD-TPase from *Mycobacterium tuberculosis,* Ldt_Mt1_ (PDB: 4JMX) with 99.5% confidence over the modeled residues, Pro98-Leu356 [11]. The structural alignment data was in agreement with the sequence alignment data with regard to the predicted catalytic triad [11]*.*

### LdtP is a periplasmic protein

**Fig. 3:**
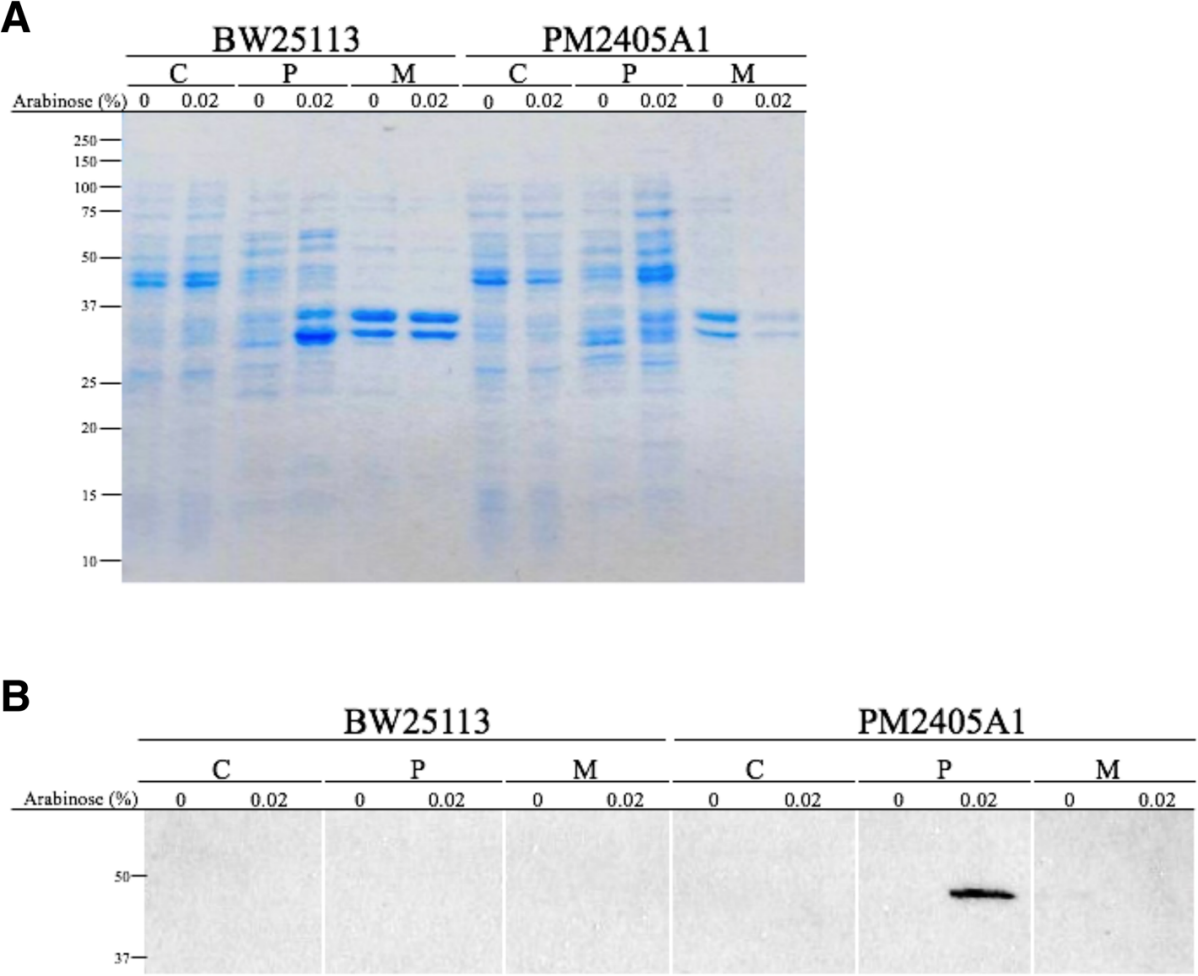
Cellular localization of LdtP. *E. coli* BW25113 and *E. coli* PM2405A1 grown in the absence and presence of 0.02% arabinose were separated into cell fractions (Panel **a**), as described, and analyzed by western blot (Panel **b**). Cytoplasmic (C), periplasmic (P), and membrane (M) fractions were probed with anti-FLAG antibody to identify the LdtP-FLAG protein

The predicted signal peptide of LdtP indicates transport of this protein into the periplasm. In order to determine the subcellular localization of LdtP, the entire 1296 bp sequence of *ldtP* was cloned into pBAD24, and the DNA sequence for a C-terminal FLAG-tag was inserted to allow for visualization via western blot. *E. coli* BW25113 (WT), PM2405 (Δldt5), and PM2405A1 (Δldt5/*ldtP*^*+*^*-FLAG*) were grown in the absence or presence of 0.02% (*w*/*v*) arabinose for induction of *ldtP* expression, and cultures were grown to OD_600_ = 1.0. Cells were fractionated into cytoplasmic, periplasmic, and membrane fractions and analyzed by western blot using an anti-FLAG antibody for detection. A single band was observed in the periplasmic fraction of PM2405A1 with a molecular weight of ~ 46 kDa, corresponding precisely with the theoretical molecular weight of LdtP (Fig. 3). No other bands were detected. These results clearly indicate that LdtP is transported to the periplasm for activity.

### *ldtP*_*Lc*_ expression is induced under osmotic stress conditions in *L. crescens*

**Fig. 4:**
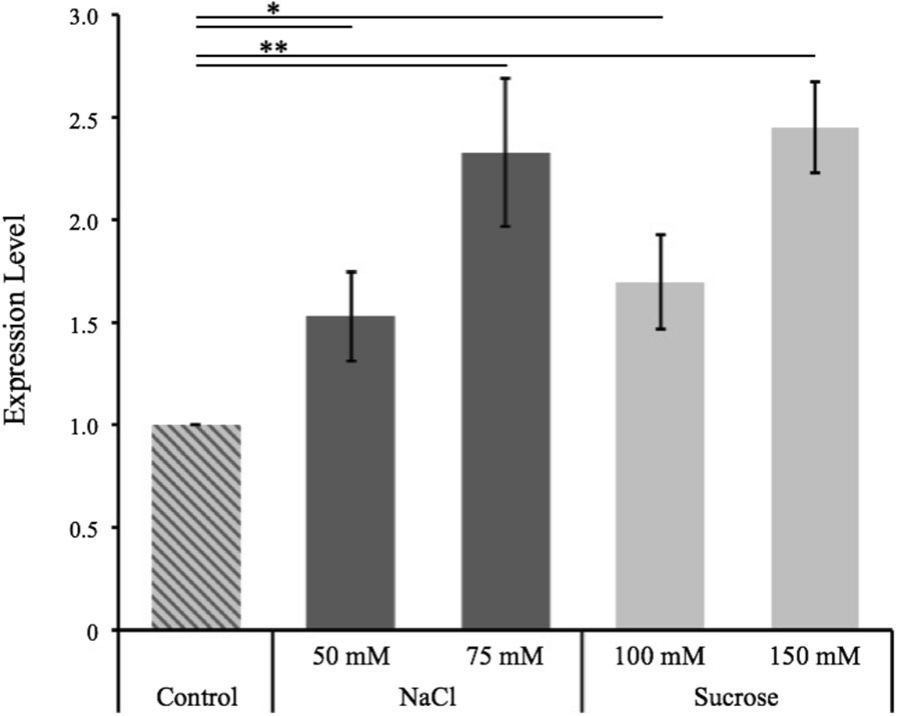
Level of expression of *ldtP*_*Lc*_ under osmotic stress conditions. The expression level of *ldtP*_*Lc*_ (*B488_10900*) was calculated against a control containing no added NaCl or sucrose and normalized with an internal standard, 16S rRNA. Values are given as the mean fold induction compared to the control with three biological replicates per condition. Statistical significance was determined using a Student’s t-test (*p < 0.05, **p < 0.01)

The expression of LdtP was previously shown to be modulated by the LdtR transcriptional regulator, a transcription factor known to regulate the expression of numerous proteins involved in the osmotic stress response [5]. LdtP was also specifically implicated as a protein critical for cell viability upon exposure to conditions with high osmolarity, such as those encountered in the phloem of the citrus tree. In order to explore this hypothesis, the level of expression of *ldtP*_*Lc*_ (*B488_10900*) was analyzed via qRT-PCR following incubation of *Liberibacter crescens* in BM7 culture media amended with non-lethal concentrations of NaCl (50 mM, 100 mM) or sucrose (75 mM, 100 mM, 150 mM). *L. crescens* is a phylogenetically close relative to *L. asiaticus* that is used as a surrogate strain due to the inability to maintain cultures of *L. asiaticus* under laboratory conditions. As expected, under our laboratory conditions, the rate of expression of LdtP increased in the presence of the high osmolarity culture media, up to 2.33 ± 0.36 fold in the presence of 100 mM NaCl and 2.45 ± 0.22 fold in the presence of 150 mM sucrose (Fig. 4). This increase in expression of LdtP correlates with the previously published results and further supports a critical role for LdtP in osmotic stress tolerance by *L. crescens*, as well as *L. asiaticus* [5].

### Complementation of *E. coli* PM2405 with LdtP results in a slow-growth phenotype

The ability of LdtP to complement a strain of *E. coli* lacking all five of the encoded LD-TPases, PM2405 (Δldt5), was investigated. The full sequence for *ldtP* was cloned into the arabinose-inducible plasmid pBAD24 generating pJFC1 [12]. PM2405A (Δldt5/*ldtP*^*+*^) was produced by transforming PM2405 (Δldt5) with pJFC1. Growth of *E. coli* BW25113 (WT), PM2405 (Δldt5), and PM2405A (Δldt5/*ldtP*^*+*^) in liquid culture was monitored under two different conditions. The assay was directed to assess the effect of LdtP overexpression on the growth kinetics of these strains. The two conditions used were (1) various arabinose concentrations beginning at *T* = 0 h and (2) cultures induced after 3 h of growth with 0, 0.02%, or 0.2% arabinose.

In the first experiment, LB broth was supplemented with increasing concentrations of arabinose (0–0.02%) before inoculation with each strain. This ensured immediate, constant induction of LdtP expression upon inoculation. The generation times for each strain at all concentrations of arabinose were calculated from the growth curve (Table 1, Additional file 2: Figure S2). When grown in the absence of arabinose, all three strains had similar duplication times (1.36, 1.44, 1.51 h, respectively). Over increasing concentrations of arabinose, the duplication times for BW25113 and PM2405 increased slightly, but none of these were statistically significant. When grown in the presence of 0.02 and 0.2% arabinose, the generation time for PM2405A increased by 148 and 126%, respectively, when compared to BW25113 at the same concentration of arabinose. These results are statistically significant in a Student’s t-test (*p* < 0.001) and indicate that the expression of LdtP in the quintuple knock-out strain causes a dramatic alteration in cell physiology and as a result, growth phenotype.

**Table 1:** Generation time of strains induced with increasing concentrations of arabinose

	BW25113		PM2405		PM2405A	
	Growth rate constant, k (generations/h)	Mean generation time (h)	Growth rate constant, k (generations/h)	Mean generation time (h)	Growth rate constant, k (generations/h)	Mean generation time (h)
0% Ara	0.74 ± 0.016	1.36 ± 0.03	0.70 ± 0.002	1.44 ± 0.01	0.66 ± 0.013	1.51 ± 0.03
0.0002% Ara	0.68 ± 0.007	1.47 ± 0.02	0.67 ± 0.010	1.50 ± 0.03	0.51 ± 0.013	1.96 ± 0.05
0.002% Ara	0.70 ± 0.006	1.43 ± 0.01	0.67 ± 0.010	1.50 ± 0.03	0.47 ± 0.027	2.11 ± 0.12
0.02% Ara	0.67 ± 0.012	1.49 ± 0.03	0.64 ± 0.009	1.56 ± 0.02	0.27 ± 0.002	3.69 ± 0.03**
0.2% Ara	0.65 ± 0.016	1.54 ± 0.04	0.63 ± 0.058	1.59 ± 0.15	0.29 ± 0.005	3.48 ± 0.07**

Generation times were calculated from the growth curves in Additional file 2: Figure S2. Values are given as mean ± SD (*n* = 3). Data were analyzed using Student’s t-test. **P-values ≤0.001 comparing mean generation time of PM2405 or PM2405A with mean generation time of BW25113 was considered statistically significant

**Fig. 5:**
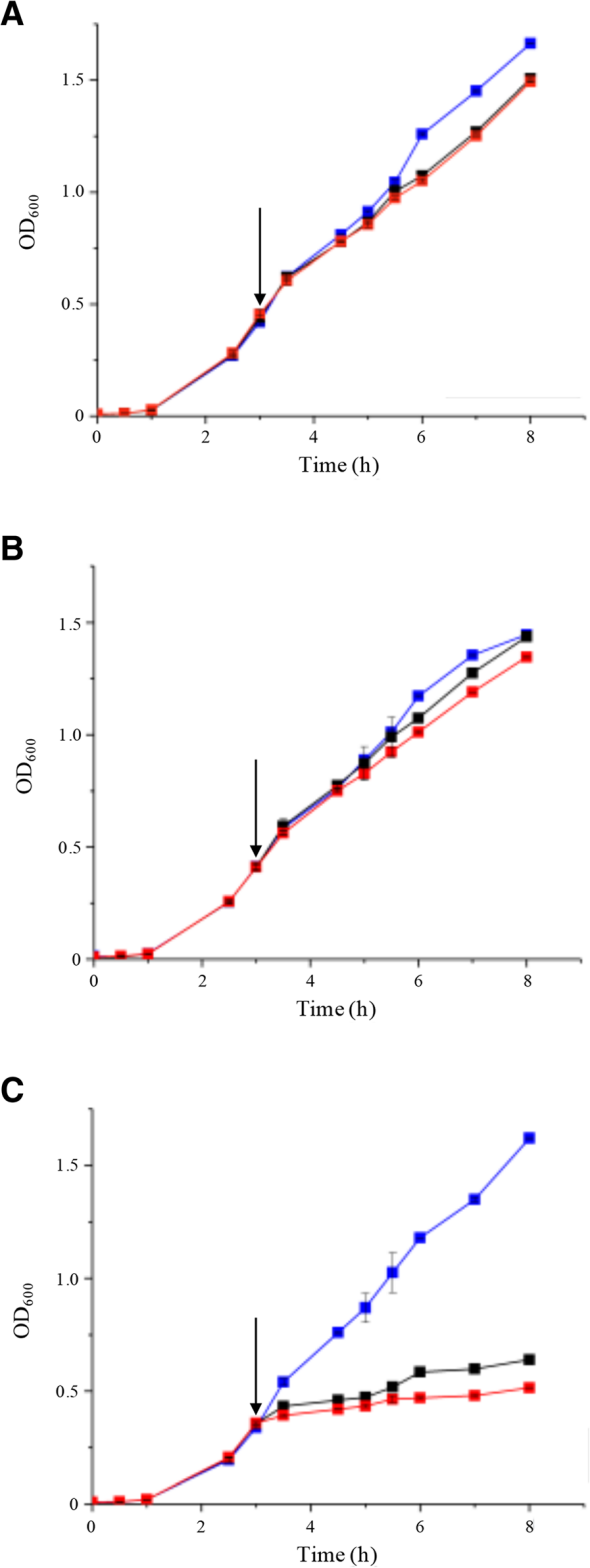
Standard growth curve upon induction of *ldtP* expression. *E. coli* BW25113 (**a**), PM2405 (**b**), and PM2405A (**c**) cultures were grown at 37 °C in Terrific broth. Cultures were induced at 3 h of growth with 0% (blue), 0.02% (black), or 0.2% (red) arabinose. Growth was monitored at OD_600_ at the indicated time points. Arrow indicates time of induction. Plotted values represent the mean ± standard deviation of three biological replicates

In the second experiment, strains were grown in terrific broth in the absence of arabinose for 3 h, at which time 0, 0.02%, or 0.2% (*w*/*v*) arabinose was added to the indicated cultures. Following induction with arabinose at either concentration, BW25113 and PM2405 showed the same growth (OD_600_) as the strains that were not induced; however, the growth of PM2405A was arrested almost immediately upon induction (Fig. 5). It can be deduced from this rapid alteration in growth phenotype that expression of LdtP results in an abrupt change in cell physiology that is so drastic as to almost completely cease cellular growth.

### Expression of LdtP results in changes in peptidoglycan cross-linking

**Fig. 6:**
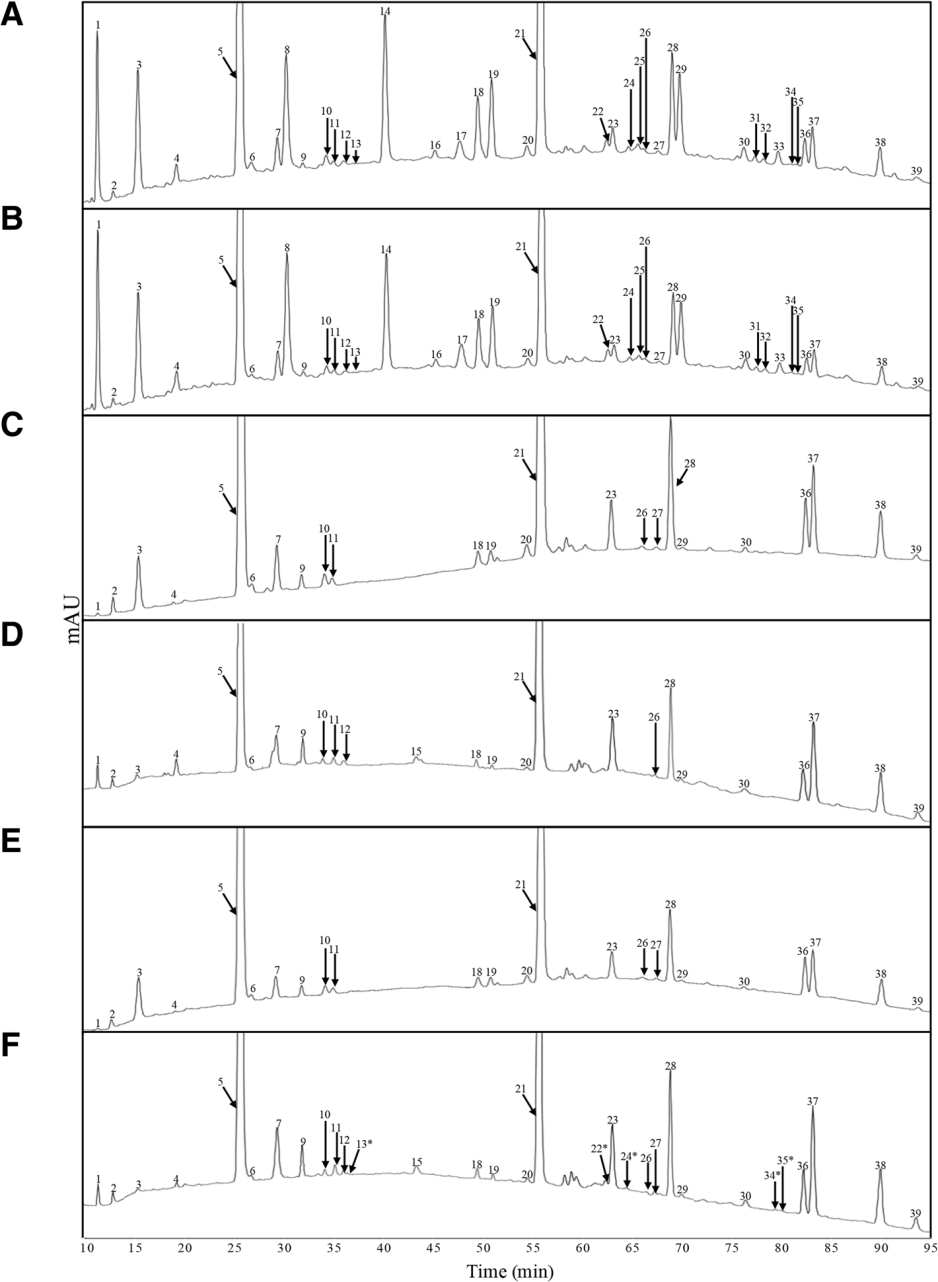
Analysis of peptidoglycan profiles. HPLC chromatograms of muropeptides acquired by digestion of peptidoglycan by mutanolysin. Peaks are numbered in accordance with Table S1. Peaks that were reintroduced in the complemented strain are indicated by asterisks. **a** *E. coli* BW25113, 0% arabinose; **b** *E. coli* BW25113, 0.02% arabinose; **c** PM2405, 0% arabinose; **d** PM2405, 0.02% arabinose; **e** PM2405A, 0% arabinose; **f** PM2405A, 0.02% arabinose

Peptidoglycan from *E. coli* BW25113 and PM2405A1 strains grown with and without arabinose was isolated, digested into muropeptides, and separated by reverse-phase high-performance liquid chromatography (HPLC). Each peak on the HPLC chromatogram (Fig. 6) corresponds to a different muropeptide. Peaks are numbered in accordance with Additional file 3: Table S1. Comparison of BW25113 and PM2405 revealed that 15 out of the 39 muropeptides disappeared with deletion of the five LD-TPases in BW25113. Disappearance of muropeptides containing a 3–3 cross-link and muropeptides linked to Braun’s lipoprotein is expected, since PM2405 does not produce the enzymes responsible for forming these cross-links [13]. Expression of LdtP in the quintuple knock-out strain, PM2405A, restored production of 5 missing muropeptides. Although the specific muropeptides affected were not identified, the reappearance of missing muropeptides clearly indicates that LdtP is involved in rearrangement of the bacterial cell wall.

### LdtP displays esterase activity in solution

A PSI-Blast multiple sequence alignment completed during the in silico analysis above, identified several amidases from different species as having high degrees of conservation to the linear protein sequence of LdtP. In vitro assays to evaluate amidase and esterase activities were performed with purified His_6X_-LdtP and model substrates, including amides, carboxyl esters (*p-*nitrophenyl esters), thioesters (acyl-CoA substrates), and aryl esters (naphthyl esters) of various chain lengths. Although LdtP showed weak or no activity toward the amides, thioesters, and aryl esters, there was substantial activity toward the short-chain carboxyl esters. The carboxyl esterase activity was stable over a range of pH from 7.0 to 8.5 and over a wide range of temperatures (30 °C to 80 °C). The saturation kinetics were demonstrated with *p-*nitrophenyl acetate and butyrate (Table 2, Additional file 4: Figure S3).

**Table 2:** Kinetic analysis of LdtP carboxyl esterase activity

	*K*_0.5_^a^ (mM)	*V*_max_^a^ (μmoles min^− 1^ mg^− 1^)	Hill coefficient (n^a^)	*k*_cat-App_ (s^− 1^)	*k*_cat-App_/*K*_0.5_ (M^− 1^ s^− 1^)
*p*NP-acetate	0.44 ± 0.035	0.67 ± 0.028	2.09 ± 0.363	0.54	1.24 × 10^3^
*p*NP-butyrate	0.44 ± 0.032	0.16 ± 0.004	3.12 ± 0.662	0.13	2.88 × 10^2^

^a^Estimated by non-linear regression analysis using MicroCal Origin 9.0

### Expression of LdtP alters lipopolysaccharide structure

**Fig. 7:**
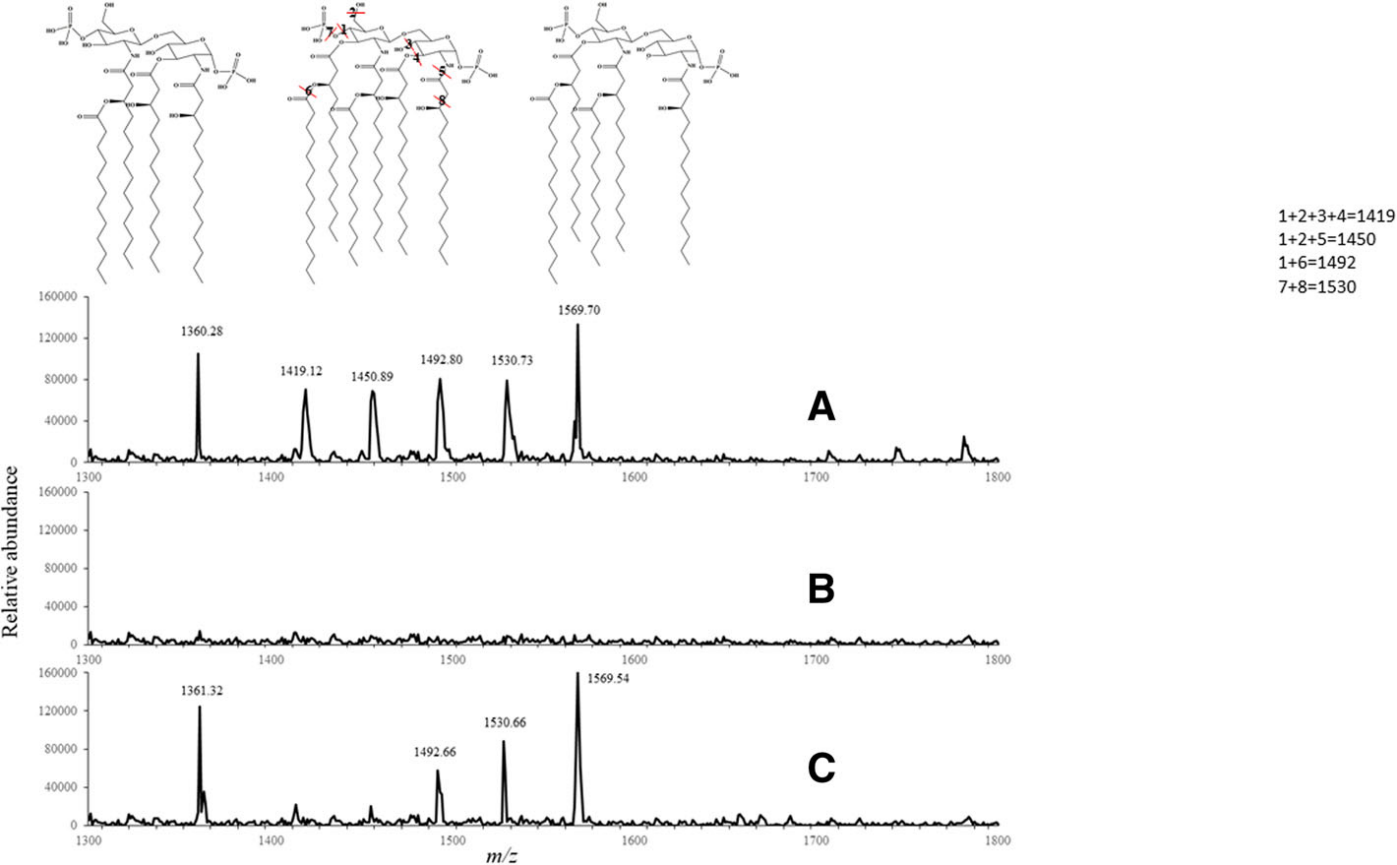
Analysis of lipid A. Mass spectrometry analysis of the lipid A fraction. **a** *E. coli* BW25113, **b** PM2405, and **c** PM2405A

Lipopolysaccharide (LPS) from *E. coli* BW25113, PM2405, and PM2405A strains grown with and without arabinose was extracted in parallel to isolate and analyze the lipid A characteristics [14, 15]. A triple quadrupole tandem mass spectrometric analysis of the lipid A fraction of each strain was carried out. Our analysis revealed low to no recovery of lipid A in the quintuple mutant. This result is probably a consequence of poor attachment of the LPS to the cell wall, most likely due to the absence of transpeptidases. The expression of LdtP in the *E. coli* quintuple mutant partially restored the lipid A in the mutant strain (Fig. 7). We followed the characteristic penta-acyl di-phosphoryl species (m/z 1360.28) and tetra-acyl di-phosphoryl species (m/z 1569.7) as biomarkers, and they were fully restored when LdtP was expressed. Four signals (m/z 1419.12, 1450.89, 1492.80, and 1530.73), presumably penta-acyl monophosphates, were also identified. Only two of those (m/z 1492.66 and 1530.66) were observed when the mutant expressing the recombinant LdtP was analyzed. Extra assays were carried out in our laboratory to fully identify the biological role of the enzyme. However, the results obtained suggest that LdtP could be involved in the modification of the LPS, likely by eliminating acyl chains with its esterase activity. The importance of the LPS structure and composition is likely a critical factor allowing the bacteria to evade plant defenses; LdtP’s putative role in altering the LPS structure identifies it as an important candidate that plays a vital role in *L. asiaticus* envelope modification and preservation in the citrus host.

## Discussion

*L. asiaticus* encounters persistent changes in osmotic pressure within the phloem of the citrus host. The soluble solid content in the phloem fluctuates among citrus species, but it may also change within a given plant depending on the season, time of day, and the specific plant tissue [5]. The phloem of the citrus tree contains sugars, amino acids, vitamins, and inorganic ions at very high concentrations [16]. Although only about 55% of the chemical composition of phloem sap is comprised of sugars, with sucrose accounting for 64% of all sugars, sucrose concentrations in phloem may vary from 15 mM up to 880 mM [16]. Thus, pathogens that reside within the phloem must have a mechanism to respond to variations in osmotic pressure. The LdtR operon from *L. asiaticus,* which includes *ldtP*, was previously characterized and identified to be involved in the osmotic stress response [5]. In agreement with this, we found that *ldtP* was strongly induced in *L. crescens* in the presence of culture media containing a high level of osmolytes, either NaCl or sucrose.

In this study, we have identified and characterized LdtP, an LD-TPase displaying esterase moonlighting activity from *L. asiaticus*. The *ldtP* gene encodes a protein with a peptidoglycan-binding domain and two YkuD LD-TPase catalytic domains that aligns with LD-TPases and amidases from other bacterial species. A structural alignment revealed the catalytic triad of LdtP and further supported the hypothesis that LdtP functions as an LD-TPase.

Due to the inability to maintain *L. asiaticus* in culture and the lack of readily available genome editing tools for *L. crescens*, the phenotypic consequences of the effects of *ldtP* expression were studied in *E. coli*. Although there is only one gene encoding an LD-TPase in *L. asiaticus*, *E. coli* has five homologs in its genome: YcfS, YnhG, YcbB, ErfK, and YbiS [7]. A strain of *E. coli* lacking all five of these LD-TPases was utilized to assess the biological effects of LdtP overproduction. Expression of LdtP in the LD-TPase mutant strain restored 5 out of the 15 missing muropeptide species represented on the HPLC chromatogram. These results suggest that *L. asiaticus* modifies its peptidoglycan structure via the action of LdtP changing the relative abundance of crosslinks, presumably 3–3 cross linking inferred from LdtP structural homology, when exposed to high osmotic stress conditions. In vitro activity assays should be performed to confirm this observation.

In bacteria, these enzymes play a pivotal role in resistance to extreme conditions. Multidrug resistance in *M. tuberculosis* is mediated by L,D- transpeptidases, carbapenems being their only inhibitor. Effective treatments against tuberculosis require simultaneous inhibition of penicillin binding proteins and L,D transpeptidases [17, 18]. L,D-transpeptidases are also used as a survival strategy by *Bdellovibrio bacteriovorus*, allowing the bacteria to resist cell wall degradation while the bacteria attacks its prey*. B. bacteriovorus* synthesizes a thicker cell wall, a phenomenon called “baiting”, which depends on the availability of substrate precursors [19–21]. A similar strategy involving identical enzymatic activity is used by *Clostridium difficile.* In this case, the terminal D-ala is replaced by D-lactate. Remarkably, this substitution decreases the affinity of glycopeptide antibiotics several fold [20–22]. The role of cell wall 3–3 crosslinked peptides is documented, but the fundamental contribution of these enzymes in bacterial pathosystems has only recently been discovered. Our previous results studying LdtR, the transcription factor that regulates *ldtP* expression, supported a similar role for this protein in *L. asiaticus* resistance [5].

Previous analyses performed in *A. tumefaciens* and *S. meliloti* indicated the presence of a highly cross-linked peptidoglycan (64%) [23]. Since only prokaryotes produce peptidoglycans, its detection by pattern recognition receptors is widespread in eukaryotic hosts (i.e., Toll-like receptor 2 and NOD receptors in mammals; peptidoglycan recognition proteins PGRPs in *Drosophila*) [24]. In plants, peptidoglycan from both gram-positive and gram-negative bacteria elicits defense responses in *Arabidopsis* [25–27]. Recently, LYM1 and LYM3 have been identified as peptidoglycan receptors in *Arabidopsis* and *Oryza sativa* (rice) [28, 29]. Notably, those genes were not among the highly expressed genes in citrus trees infected with *L. asiaticus*. We hypothesize that LdtP could be involved in modifications of the peptidoglycan structure as a means of “hiding” the bacterium from the host immune system. Such modifications will favor *L. asiaticus* persistence within the host while the bacteria evade detection and elimination by the plant immune system.

Analysis of the lipid A moiety of the LPS in the various *E. coli* strains investigated showed alterations in structure. The *E. coli* quintuple mutant LD-TPase strain expressing LdtP showed a different pattern of lipid A composition compared to the wild type. This result, along with the demonstrated esterase activity in vitro*,* supports an additional role for LdtP in the modification of the cell LPS. LPS is extremely heterogeneous; for example, in *Rhizobium* species, the specific modifications in structure can vary in response to external stimuli, such as environmental conditions [30]. LPS is essential in symbiotic bacteria and is a strong immune response activator in pathogenic bacteria [31, 32]. Modifications of the LPS structure, specifically in the lipid A region, have been shown to have dramatic effects on bacterial survival and virulence and can alter host immune responses [33]. Modifications of *L. asiaticus* lipid A by LdtP, probably due esterase activity, may also contribute to the survival strategy of this bacterium, allowing it to thrive in the phloem of the citrus host.

The moonlighting activity proposed for LdtP, based on the esterase activity and modification of LPS, is the first report associating such activity with a cell wall crosslinking enzyme. This dual job is likely a consequence of a drastic evolutionary reduction of the genetic information encoded in *L. asiaticus*’ genome. The intracellular lifestyle of this bacteria and genomic reduction has allowed for fewer encoded enzymes required to fulfill all basic cellular needs.

Transcriptional activation of *ldtP* was crucial to increase osmotic stress tolerance in a phylogenetically-related surrogate species, *S. meliloti*, during assay conditions [5]. This suggests that the activity of LdtP may be essential to the survival of *L. asiaticus* in response to the high osmotic pressure sustained within the citrus phloem by increasing the rigidity of the cell wall through an increase in the abundance of 3–3 cross-links. Alternatively, *L. asiaticus* may also mask the peptidoglycan and LPS from detection by the citrus plant immune system by modification that improves viability and survival within the host.

## Conclusions

These data confirm that LdtP is involved in the osmotic stress response and indicate LdtP’s likely role in *L. asiaticus* cell wall rearrangement that may promote the persistence of this bacterium in the phloem of citrus plants. Targeting LdtP for the development of therapeutic agents against HLB is an approach that will have a significant effect on survival of this pathogen in the host.

## Materials and methods

### Chemicals

All restriction enzymes, RNase H (5000 U mL^− 1^), and DNase I (2000 U mL^− 1^) were purchased from New England Biolabs, Inc.. Halt™ protease & phosphatase inhibitor cocktail, EDTA-free (100X) was purchased from Thermo Scientific. Brain heart infusion was purchased from Difco Laboratories. L-(+)-arabinose, α-amylase from *Aspergillus oryzae* (30 U mg^− 1^), protease from *Streptomyces griseus* (Pronase E; ≥ 3.5 U mg^− 1^), mutanolysin from *Streptomyces globisporus* ATCC 21553 (≥ 4000 U mg^− 1^), sodium azide, all antibiotics, and culture media components were purchased from Sigma-Aldrich. All other chemicals were of analytical grade.

### Bacterial strains and growth conditions

Bacterial strains and plasmids are listed in Table 3. *Escherichia coli* strains were grown in Luria-Bertani (LB) broth, LB agar, Terrific (TB) broth, and M9 minimal medium at 37 °C, as indicated. When required, the media was supplemented with ampicillin (100 μg mL^− 1^) and kanamycin (50 μg mL^− 1^). Gene expression was induced with arabinose, as indicated.

**Table 3:** Bacterial strains and plasmids used in this study

Name	Relevant genotype	Reference
Bacterial Strain		
*L. crescens* BT-1	Standard strain	Leonard et al. (2012) [34]
*E. coli* DH5α	φ80 d*lacZ*ΔM15Δ(*lacZYA-argF*)U169 *recA1 endA1 hsdR17* (rk^−^. mk^+^) *supE44 thi-1 gyrA relA1*	Laboratory stock
E. coli BL21 (DE3)	*F− ompT gal dcm lon hsdSB(rB- mB-) λ(DE3 [lacI lacUV5-T7 gene 1 ind1 sam7 nin5])*	Life Technologies
*E. coli* BW25113	Δ(*araD-araB*)*567* Δ*lacZ4787*(::rrnB-3) λ^−^ *rph-1* Δ(*rhaD-rhaB*)*568 hsdR514;* (WT)	Kitagawa et al. (2005) [48]
JC1001	*E. coli* BW25113 pJFC1*;* (Δ*ldt5/ldtP*^*+*^)	This study
PM2405	*E. coli* BW25113 Δ*ynhG753*::frt Δ*ybiS790*::frt Δ*ycfS775*::frt Δ*erfK761*::frt Δ*ycbB742*::*kan;* (Δ*ldt5*)	Sanders & Pavelka (2013) [7]
PM2405A	*E. coli* PM2405 pJFC1*;* (Δ*ldt5/ldtP*^*+*^)	This study
PM2405A1	*E. coli* PM2405 pJFC1F*;* (Δ*ldt5/ldtP*^*+*^ *-FLAG*)	This study
Plasmid		
p15TV-L	Expression vector for purification of proteins by nickel affinity chromatography. Ap^R^	Structural Genomics Consortium, University of Toronto
pBAD24	Arabinose-inducible *araBAD* expression vector; optimized Shine-Dalgarno box (RBS), no purification tag in MCS; Ap^R^	Guzman et al. (1995) [12]
pJFC1	pBAD24 derivative containing *ldtP* from *L. asiaticus* (NcoI/AccI); Ap^R^	This study
pJFC1F	pBAD24 derivative containing *ldtP* from *L. asiaticus* with a C-terminal FLAG-tag (NcoI/AccI); Ap^R^	This study

*L. crescens* BT-1 was cultured at 25 °C with moderate agitation (200 rpm) in modified BM7 media, pH 6.9, as described previously [5, 34]. This culture media was composed of 1% Brain Heart Infusion, 15% Fetal Bovine Serum, 30% TMN-FH insect medium, α-Ketoglutaric acid (2 mg mL^− 1^), ACES (10 mg mL^− 1^), and potassium hydroxide (3.75 mg mL^− 1^).

### DNA manipulations

Primers are listed in Table 4. Standard methods were used for cloning, ligation, and transformation [35]. PCR products were purified using Qiaquick purification kits (Qiagen), and plasmids were isolated using QIAprep Spin Miniprep Kit (Qiagen).

**Table 4:** Primers used in this study

Primer	Oligonucleotide Sequence (5′ → 3′)
Protein Purification	
LdtP_Ext_Fw	GGGGTGATCAGATTGCGTAT
LdtP_Ext_Rv	ATCCTTGCGCCTCTAAAACA
LdtP_LIC_Fw	TTGTATTTCCAGGGCGTAGAGAAACCCATTCATGC
LdtP_LIC_Rv	CAAGCTTCGTCATCAGTCAGAATCTATAGGATGATCCT
pBAD24 Cloning	
LdtP_NcoI_Fw	CGATTAGCCATGGTTGGATATTTAAAGATAAATAAG
LdtP_AccI_Rv	TTTATTTAGTCGACGATCAGTCAGAATCTATAG
pBAD24-LdtP_C-terminalFLAG_Fw	gatgatgataaaTGATCGTCGACCTGCAGG
pBAD24-LdtP_C-terminalFLAG_Rv	atctttataatcGTCAGAATCTATAGGATGATCCTC
Sequencing	
T7-Promoter	TAATACGACTCACTATAGGG
T7-Terminator	GCTAGTTATTGCTCAGCGG
pBAD-Forward	ATGCCATAGCATTTTTATCC
pBAD-Reverse	GATTTAATCTGTATCAGG
qRT-PCR	
B488_16S_Fw	CAGAACCTTACCAGCCCTTG
B488_16S_Rv	ATTAGCTCCGCCTCACGACT
B488_10900_Fw	GGTGGCCACAGGTGGTAATA
B488_10900_Rv	TAACCCATGTCGTGCTTGAA

For protein expression and purification, the *ldtP* gene was amplified from *L. asiaticus* str. psy62 chromosomal DNA by PCR and then cloned into the p15TV-L plasmid (GenBank accession EF456736; Structural Genomics Consortium, Toronto) as described previously [36].

Cloning in the pBAD24 plasmid was performed by amplifying *ldtP* from *L. asiaticus* str. psy 62 chromosomal DNA by PCR, using the primers in Table 4. The PCR product was verified by agarose gel electrophoresis, and both the PCR product and the pBAD24 plasmid were digested with NcoI and AccI in a simultaneous double digestion reaction according to the manufacturer’s instructions. A ligation reaction was performed with the Quick Ligation™ Kit (New England Biolabs, Inc.). Insertion of a FLAG-tag sequence at the C-terminus of the *ldtP* gene in the pBAD24 vector was completed by PCR with the primers listed in Table 4 and the Q5® Site-Directed Mutagenesis Kit (New England Biolabs, Inc.). Clones were confirmed by sequencing using pBAD universal primers.

### Protein purification

The overexpression of a soluble His-tagged LdtP fusion protein (residues 27–431) in *E. coli* BL21-Star(DE3) cells (Life Technologies) was performed exactly as described previously [4, 5].

Protein purification was also performed as described previously by Pagliai et al. [4] but with minor adjustments to the purification buffers. The binding buffer, wash buffer, and elution buffer were all composed of 500 mM NaCl, 5% glycerol, 50 mM Hepes pH 7.5, and 0.5 mM TCEP; however, the imidazole concentration differed in each at 5 mM, 0.5 mM, and 250 mM, respectively. The purified protein was dialyzed against 250 mM NaCl, 5% glycerol, 10 mM Hepes pH 7.5, 0.5 mM TCEP at 4 °C overnight. The final protein concentration was determined using the Bio-Rad protein assay (Bio-Rad), before the purified protein was aliquoted and stored at − 80 °C.

### Size-exclusion chromatography

Size exclusion chromatography with a Superose 12,100/300 GL gel filtration column (GE Healthcare) was completed as described by Pagliai et al. [4].

### Cell fractionation

Cells were fractionated into cytoplasmic, periplasmic, and membrane fractions as described previously, with some modifications [37, 38]. Briefly, cells were grown in 100 mL cultures to an OD_600_ = 1.0, collected by centrifugation, and gently resuspended in 1 mL of TSE buffer (200 mM Tris-HCl pH 8.0, 500 mM sucrose, 1 mM EDTA, 1X protease & phosphatase inhibitor), pre-chilled to 4 °C [38]. The cell suspension was incubated on ice for 30 min to allow for the complete release of periplasmic proteins. Centrifugation at 16,000×*g* for 30 min at 4 °C separated the periplasmic fraction (supernatant) from the spheroplasts, which contain the cytoplasmic and membrane fractions. The pelleted spheroplasts were resuspended in 750 μL of sonication buffer (50 mM Tris-HCl pH 7.5, 50 U RNase H, 40 U DNase I, 2.5 mM MgCl_2_, 0.5 mM CaCl_2_, 75 mM KCl), incubated for 5 min on ice, and lysed by sonication. Cellular debris was removed by centrifugation (3000×*g*, 15 min, 4 °C). The supernatant, containing the cytoplasmic and membrane fractions, was ultracentrifuged at 300,000×*g* for 2 h at 4 °C to further separate the fractions. The supernatant contained the cytoplasmic fraction, and the pellet, containing the membrane fraction, was resuspended in membrane protein extraction buffer (50 mM Tris-HCl pH 8.0, 2% Triton X-100, 10 mM MgCl_2_). All fractions were aliquoted and stored at − 80 °C.

### Western blot

SDS-PAGE of cell fractions was performed according to the method described by Laemmli (1970) [39]. Cell fraction samples were prepared by incubation at 100 °C for 5 min and separated on 12.5% polyacrylamide gels. Proteins were transferred to Amersham Hybond™-N^+^ nylon hybridization transfer membranes (GE Healthcare) at 450 mA for 40 min in a semi-dry transfer and then probed with mouse polyclonal anti-FLAG antibody (Sigma-Aldrich). The membranes were then incubated with rabbit anti-mouse antibody coupled to horseradish peroxidase. The presence of LdtP was detected by chemiluminescence using the Amersham ECL™ western blotting detection reagents (GE Healthcare) according to the manufacturer’s instructions.

### qRT-PCR

*L. crescens* BT-1 cells were cultured at 200 rpm in BM7 culture media amended with NaCl (50 mM, 100 mM) or sucrose (75 mM, 100 mM, 150 mM) at 25 °C. The cells were collected by centrifugation at 4 °C when the cultures reached mid-exponential phase (OD_600_ = 0.3). Total RNA was extracted with the RiboPure-Bacteria kit (Life Technologies), and cDNA was synthesized using the iScript cDNA synthesis kit (Bio-Rad).

qRT-PCR assays were carried out in a Bio-Rad iCycler IQ apparatus, using the iQ SYBR Green SuperMix (Bio-Rad). The changes of expression (C_t_ values) between the samples treated with NaCl or sucrose compared to the control were determined using the 2^-ΔΔCt^ method. The sequence of primers for B488_10900 and 16S rRNA, used as an internal control, are listed in Table 4.

### Growth curve assay and generation time calculation

*E. coli* strains were grown in LB or Terrific broth supplemented with increasing concentrations of arabinose (0–0.2%). The optical density (600 nm) of the cultures was recorded hourly, and a plot containing the log(OD_600_) versus time was created with Origin 9.0 software (OriginLab) to facilitate the calculation of the growth rate constant (*k*) and the generation time (*g =* 1/*k*) as previously described [4]. The assays were performed in triplicates, and statistical significance was determined using a Student’s t-test.

### Purification and digestion of the peptidoglycan sacculus

*E. coli* strains were grown in 250 mL of M9 broth at 37 °C to late exponential phase, approximately to OD_600_ = 0.6. Peptidoglycan was purified as described previously with a few modifications [40–42]. The cultures were rapidly chilled in an ice/ethanol bath before the cells were collected by centrifugation at 12,000×*g* for 15 min at 4 °C. The cells were washed once with 20 mM sodium acetate buffer, pH 5.0, pre-chilled to 4 °C, and resuspended in 5 mL of the same buffer [41]. The cell suspension was added dropwise to 5 mL of boiling 10% SDS (100 °C) and incubated at this temperature with continual stirring for 3 h. After this incubation, the heat was turned off, and the cells continued to stir overnight at room temperature. Insoluble peptidoglycan sacculi were collected by ultracentrifugation at 400,000×*g* for 15 min at room temperature, washed three times with ultrapure water, and resuspended in 800 μL of Buffer A (10 mM Tris-HCl, pH 7.2 + 0.06% (*w*/*v*) NaCl) [40].

To eliminate high-molecular weight glycogen, the sacculi were treated with 200 μL of 1 mg mL^− 1^ α-amylase in Buffer A (200 μg mL^− 1^ final concentration) and incubated at 37 °C for 1 h. To remove covalently-bound lipoprotein, the sacculi were treated with 200 μL of 1 mg mL^− 1^ pre-activated Pronase E in Buffer A (200 μg mL^− 1^ final concentration) and incubated at 60 °C for 2 h. Digestions were stopped by the addition of 200 μL of 6% SDS followed by boiling at 100 °C for 5 min. Sacculi were collected by ultracentrifugation at 400,000×*g* for 15 min at room temperature, washed three times with ultrapure water to remove residual SDS, and resuspended in 200 μL of 50 mM sodium phosphate buffer, pH 6.2.

Sacculi were digested to muropeptides by treating the samples with mutanolysin from *Streptomyces globisporus* ATCC 21553 at a final concentration of 40 μg mL^− 1^ at 37 °C overnight. The samples were boiled at 100 °C for 5 min to stop the digestion, and soluble muropeptides were recovered from the supernatant after centrifugation at 16,000×*g* for 10 min at room temperature. Before analysis via HPLC, muropeptides were reduced for 30 min at room temperature by adding an equal volume of 500 mM borate buffer, pH 9.0, and sodium borohydride to a final concentration of 10 mg mL^− 1^. The final pH of the samples was adjusted to between 3.0–4.0 with phosphoric acid. Samples were aliquoted and stored at − 80 °C.

### High-performance liquid chromatography (HPLC) and mass spectrometry

Reverse-phase HPLC was used to quantify the relative abundance of individual muropeptide species in order to facilitate meaningful comparisons between bacterial strains. A Hitachi LaChrom Elite HPLC system consisting of two L-2130 pumps, an L-2200 autosampler, an L-2300 column oven, and an L-2400 UV detector was used (Hitachi). Samples were passed through a 0.22 μm filter, and injection volumes of 30 μL were separated by HPLC on a Hypersil ODS column (250 × 4.6 mm, 3 μm; Thermo Scientific) using a linear gradient from 50 mM sodium phosphate, pH 4.31, containing 1 μg mL^− 1^ sodium azide to 75 mM sodium phosphate, pH 4.95, containing 15% methanol over 135 min at 55 °C with a flow rate of 0.5 mL min^− 1^ [42]. UV detection was performed at 205 nm. Data were analyzed with EZChrom Elite 3.3.2 software (Agilent). The abundance of each muropeptide was determined as the peak area percentage of the total integrated peak area [6, 43]. Mass spectrometry: a TSQ Quantum Access MAX Triple Quadrupole tandem mass spectrometric analysis system (Thermo Scientific, San Jose, CA, USA) was carried out using a heated-electrospray ionization in negative mode (HESI-) in this study. The ion spray voltage, capillary temperature, and collision energy were set at 3500 kV, 300 °C, and − 10 eV, respectively. The samples (10 μg mL^− 1^ in 50% ethanol) were injected into the instrument by a syringe pump, and the flow rate of the syringe pump was set to 10 μL min^− 1^.

### Esterase and amidase activity assay

Carboxyl esterase, aryl esterase, and thioesterase activities were assessed using *p-*nitrophenyl esters, naphthyl esters, and acyl-CoA substrates, respectively, and activity was measured spectrophotometrically by continuous reading at 400 nm (ɛ = 10,500 M^− 1^ cm^− 1^), 310 nm (ɛ = 3000 M^− 1^ cm^− 1^), or 412 nm (ɛ = 13,600 M^− 1^ cm^− 1^). The activity assays were carried out as described previously [44–46]. All assays were conducted at 30 °C, and the reactions were started by adding purified His_6X_-LdtP at a final concentration of 20 μg mL^− 1^. Control reactions, with the addition of buffer in the place of enzyme, were used to estimate any auto-hydrolysis of the substrate. The kinetic parameters were determined by plotting the initial velocities over increasing initial substrate concentrations and generating curves fitted with the Hill equation using MicroCal Origin 9.0 (OriginLab).

Amidase activity was measured via an Ammonia Assay Kit (Sigma-Aldrich), as the release of ammonia follows hydrolysis of an amide bond. Amides with various acyl chain lengths were tested as substrates. The reactions were carried out at 30 °C, and the reactions were started by adding purified His_6X_-LdtP at a final concentration of 20 μg mL^− 1^. Control reactions, with the addition of buffer in the place of enzyme, were used to estimate any auto-hydrolysis of the substrate. The concentration of resulting ammonia was measured according to the manufacturer’s protocol.

### LPS extraction and lipid a isolation

*E. coli* strains were grown in 250 mL of LB broth with or without 0.02% arabinose at 37 °C to late exponential phase, approximately to OD_600_ = 0.6, and lyophilized. LPS was extracted as described previously with a few modifications [14]. Lyophilized cells were weighed out to 120 mg each, resuspended in 5 mL of TRI Reagent (Sigma-Aldrich), and incubated at room temperature for 15 min to lyse the cells. Following incubation, 2.4 mL of chloroform was added, and the samples were vigorously vortexed and incubated at room temperature for 10 min to facilitate a phase separation. The samples were centrifuged at 12,000 x *g* for 10 min to separate the phases, and the aqueous phase was transferred to a new tube. Three water extraction steps were completed to ensure complete removal of LPS from the organic phase; the aqueous phases from each step were pooled and lyophilized.

Lipid A was isolated from whole LPS via mild acid hydrolysis as described by Yi and Hackett [14]. Briefly, lyophilized LPS samples were resuspended in 1% SDS in 10 mM sodium acetate (pH 4.5) and incubated in an ultrasound bath to completely dissolve the sample. The samples were then heated at 100 °C for 1 h, lyophilized, and washed twice with acidified ethanol to remove the SDS. Isolated lipid A was lyophilized and stored at − 80 °C.

The lipid A was visualized by Tris-Tricine SDS-PAGE followed by silver staining as described by Schägger [15].

## Additional files

Additional file 1: **Figure S1.** SDS-PAGE showing purification of recombinant His-tagged LdtP. (TIF 347 kb)

Additional file 2: **Figure S2.** Growth curves for *E. coli* BW25113, PM2405, and PM2405A with various concentrations of arabinose. (TIF 269 kb)

Additional file 3: **Table S1.** Peak areas of HPLC-separated muropeptides isolated from *E. coli* strains. Peak areas were calculated as a percentage of the total integrated area and are numbered as labeled in Fig. 6. *Peaks that were reintroduced in the complemented strain. (XLS 33 kb)

Additional file 4: **Figure S3.** Saturation kinetics for *p*-nitrophenyl acetate and butyrate. (TIF 173 kb)

## Abbreviations

DD-TPase: D,D-transpeptidase
LD-TPase: L,D-transpeptidase
PG: Peptidoglycan
