# Supplementary figures and images for: Purification and partial characterization of LdtP, a cell envelope modifying enzyme in Liberibacter asiaticus
Source: BMC Microbiol. 2018 Nov 29;18:201. doi: 10.1186/s12866-018-1348-8 (PMC6267092; doi:10.1186/s12866-018-1348-8)

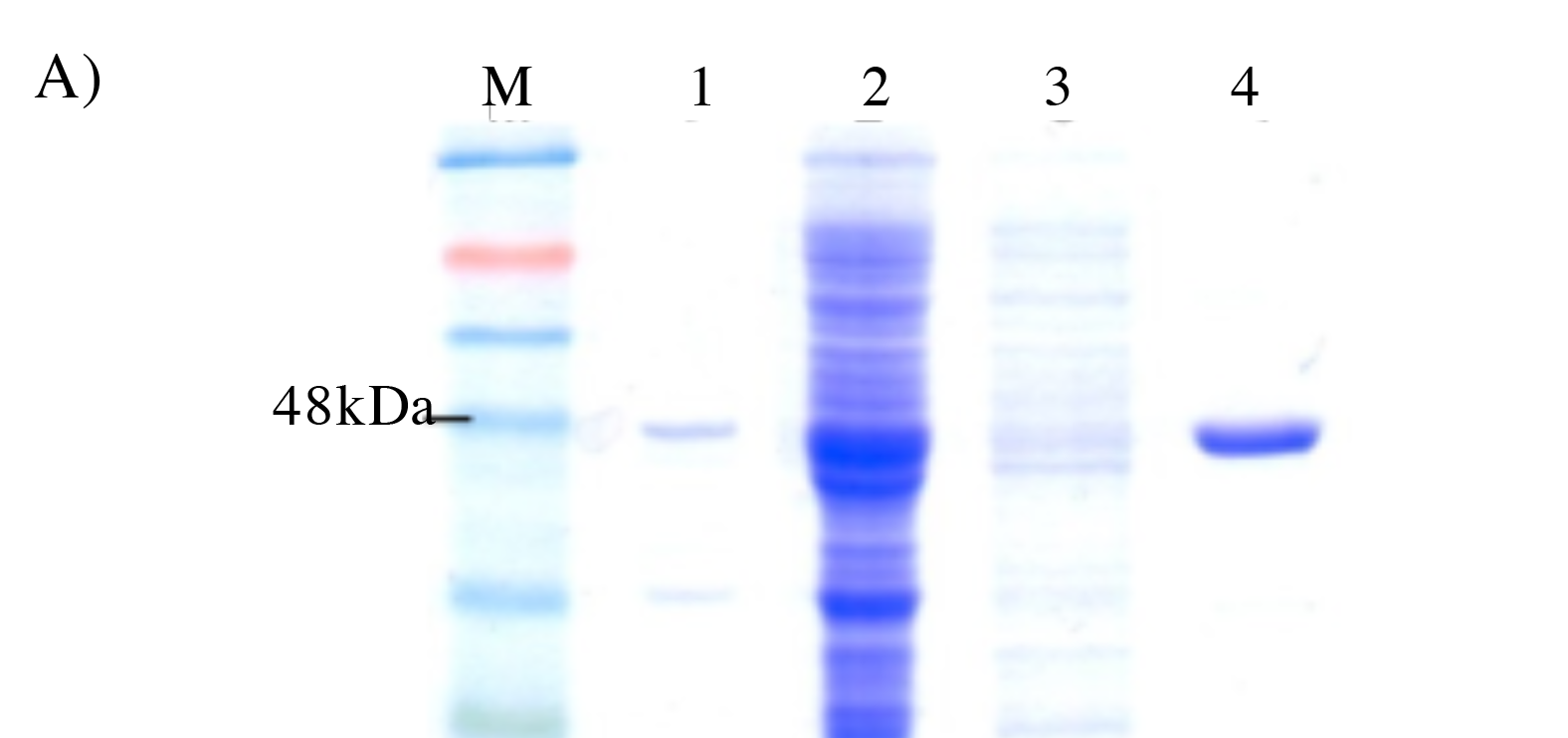

Supplement: Supplementary file 1 — Figure S1. SDS-PAGE showing purification of recombinant His-tagged LdtP. (TIF 347 kb) [file 12866_2018_1348_MOESM1_ESM.tif]

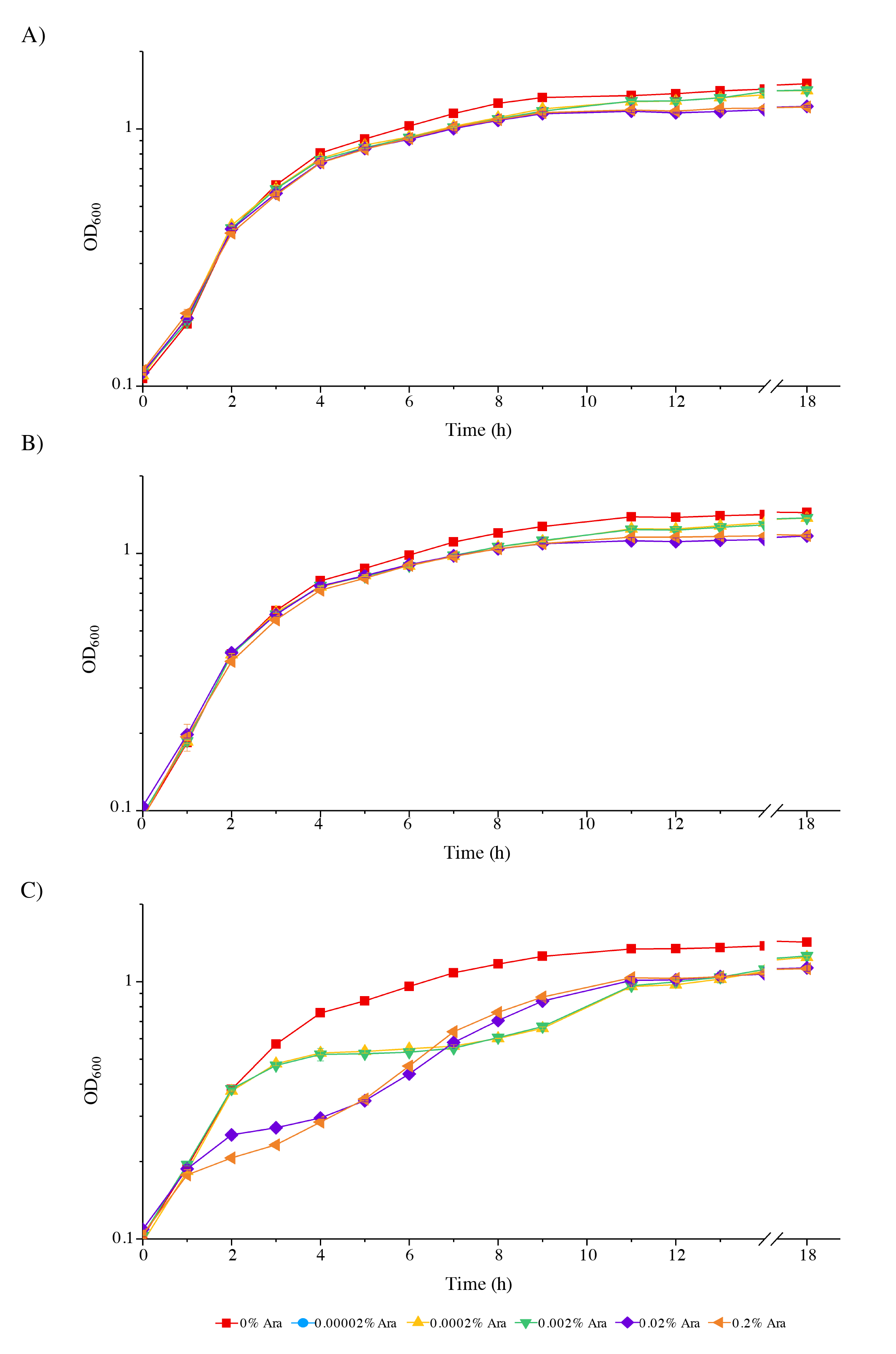

Supplement: Supplementary file 2 — Figure S2. Growth curves for E. coli BW25113, PM2405, and PM2405A with various concentrations of arabinose. (TIF 269 kb) [file 12866_2018_1348_MOESM2_ESM.tif]

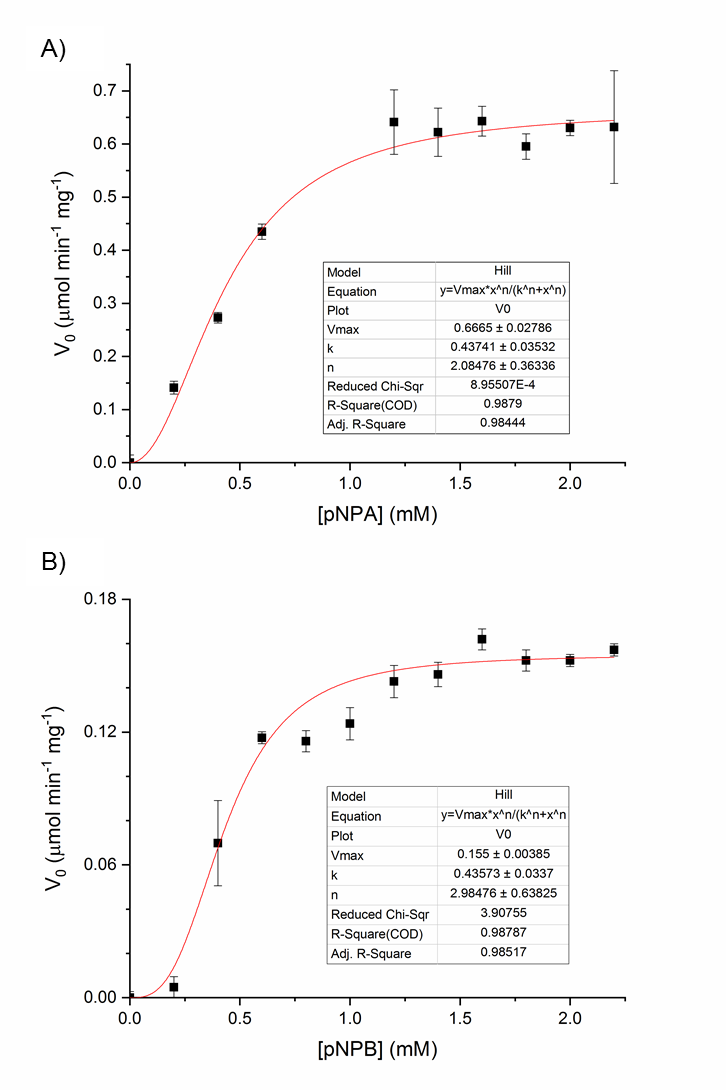

Supplement: Supplementary file 4 — Figure S3. Saturation kinetics for p-nitrophenyl acetate and butyrate. (TIF 173 kb) [file 12866_2018_1348_MOESM4_ESM.tif]
